# Lignin degradation in corn stalk by combined method of H_2_O_2_ hydrolysis and *Aspergillus oryzae* CGMCC5992 liquid-state fermentation

**DOI:** 10.1186/s13068-015-0362-4

**Published:** 2015-11-19

**Authors:** Zhicai Zhang, Lili Xia, Feng Wang, Peng Lv, Maxiaoqi Zhu, Jinhua Li, Keping Chen

**Affiliations:** Institute of Agro-production Processing Engineering, Jiangsu University, Zhenjiang, 212013 People’s Republic of China; Beijing Green Technology and Natural Biotechnology Co., Ltd., Beijing, 102300 People’s Republic of China; Institute of Life Science, Jiangsu University, Zhenjiang, 212013 People’s Republic of China

**Keywords:** Biofuel, Lignin peroxidase, Sugar yield, Hydrolysis, Fermentation, Corn stalk

## Abstract

**Background:**

Lignin peroxidase (LiP) is the primary enzyme responsible for lignin degradation. In our previous work, in order to shorten the pretreatment time and increase the lignin degradation, we have pretreated the corn stalk (CS) using a combination of *Aspergillus oryzae* CGMCC 5992 solid-state fermentation and H_2_O_2_ treatment.

**Results:**

In the present study, one-factor-at-a-time design and response surface design were applied to optimize the nutritional constituents for LiP production in liquid-state fermentation by *A. oryzae* CGMCC 5992 and the conditions for CS degradation by *A. oryzae* CGMCC 5992. The optimal medium included CS of 30 g/L, glucose of 4.6 g/L, sodium nitrate of 1.2 g/L, corn steep liquor of 1 g/L, yeast extract of 1.2 g/L, and vitamin B_1_ of 0.15 g/L. Under these optimal conditions, the LiP production reached its maximum of 652.34 U/L. The optimal condition for CS degradation included CS of 20 g, *A. oryzae* CGMCC 5992 broth of 50 mL, 1.5 % H_2_O_2_ solution of 80 mL, H_2_O_2_ flow rate of 0.4 mL/min, water volume of 240 mL (water/material ratio of 12:1), hydrolysis temperature of 39 °C, and hydrolysis time of 8 h. Before hydrolysis, CS and water were pretreated at 113 °C for 11 min. Under these optimal conditions, the sugar yield reached its maximum of 46.28 %.

**Conclusions:**

Our newly developed method had great advantages in pretreatment of CS due to its quickness, convenience, safety, no special equipment and high sugar yield.

**Graphical abstract:**
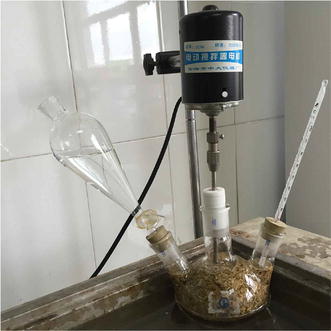
The schematic diagram of corn straw hydrolysis

**Electronic supplementary material:**

The online version of this article (doi:10.1186/s13068-015-0362-4) contains supplementary material, which is available to authorized users.

## Background

Lignocellulosic materials account for the major portion of agricultural residues on earth. Among the agricultural products, straws from cereal crops are the major by-product from agricultural fields in China. According to statistics provided by the government of China, corn output reached 2.88 × 10^8^ tons and the by-product of corn straw was about 2.55 × 10^8^ tons in 2014 [1]. Common practices for agricultural waste management include open field burning, cattle breeding, landfill, and inclusion in the household waste collection. In many regions, most of the cereal straws are just retained on the fields or incorporated into the soil. Therefore, crop stalk represents an abundant, cheap, and readily available source for the biofuel production.

Lignin, hemicellulose, and cellulose are the three major chemical components of corn stalk (CS). As a macromolecule and highly branched polymer, lignin forms the lignin sheath and surrounds hemicellulose and cellulose, which protects the cellulose and hemicellulose from degradation by cellulase and hemicellulase [2–4]. The crystal structures of cellulose and hemicelluloses cause difficulties to fully swell like starch in the process of high-temperature pretreatment, leading to the reduced access surface areas and the limited degradations of cellulase and hemicellulase. Therefore, it is necessary to remove lignin from biomass and destroy such crystal structure in order to convert biomass resources (such as CS) into industrial products (such as biofuel).

Based on physicochemical technologies, different methods have been developed to degrade lignin and destroy the crystal structure of cellulose in ligno-cellulosic substrates, such as microwave, ionizing radiation, steam explosion, acid or alkali dilution, and oxidation or a variety of their combinations [5]. However, these typical physical and chemical techniques require not only high-energy (steam or electricity) but also corrosion-resistant and high-pressure reactors, resulting in increased demand of special equipments and cost of pretreatment as well as pollution, such as organic waste water and exhaust gas emission [6, 7].

Lignin can be degraded by extracellular enzymes from microorganisms, including lignin peroxidase (LiP), laccase (Lac), manganese peroxidase (MnP), versatile peroxidase (VP) [8], and dye-decolorizing peroxidases (DyP) [9]. As one of the key enzymes in the extracellular lignin degradation system, LiP consists of a single polypeptide chain with an iron protoporphyrin prosthetic group. Moreover, it has a unique ability to degrade lignin polymer through an oxidative electron transfer mechanism [10]. It is believed that the white rot fungi can be used for LiP production and lignin degradation. However, fungal pretreatment has four major drawbacks, including the relatively low efficiency, considerable carbohydrate loss, large cover area, and long fermentation cycle. Therefore, it is necessary to screen new strains and explore new methods in order to overcome these shortcomings.

*Aspegillus oryzae* has been reported to secrete many types of enzymes, including protease, amylase, cellulase, and phytase [11]. However, only few papers have reported the ability of *A. oryzae* to synthesize hydrolytic enzymes of lignin. In our previous study, we have isolated a strain in the sludge of the Yudai River in Jiangsu University, and found that it can remove COD from vinasse [12]. This strain has been identified as *A. oryzae* CGMCC 5992 according to its morphology and 28 s rDNA sequence. Furthermore, gallic acid is used as the substrate, and the activity of various enzymes related with its degradation has been analyzed. It has been found that the strain secrets LiP, MnP and Lac in the process of degradation of gallic acid [13]. The proteomic analysis has shown that the strain secretes LiP, endo-1,4-β-d-glucanase and alkaline proteinase, and neutral proteinase in the presence of CS [14], suggesting that these enzymes play key roles in the degradation of lignin and aromatic compounds [15].

As a broad-spectrum sterilizing agent, H_2_O_2_ can be used as a restrictive substrate of the hydrolytic reaction catalyzed by LiP and MnP. Therefore, pretreatment of CS with H_2_O_2_ not only sterilizes other bacteria and fungi, but also provides the necessary substrate for the hydrolytic reaction of lignin. We have studied the possibility of using *A. oryzae* CGMCC 5992 to degrade lignin of CS pretreated with different concentrations of H_2_O_2_ in the solid-state fermentation, and we have found that *A. oryzae* can grow well on CS pretreated with 3 % H_2_O_2_. H_2_O_2_-pretreated CS has shown higher synthesis of MnP and LiP and higher disintegration of lignin, but it inhibits cellulase synthesis and cellulose degradation [16]. Moreover, combination of *A. oryzae* CGMCC 5992 solid-state fermentation and H_2_O_2_ hydrolysis has been applied in pretreatment of CS. Although such a combination method can shorten the treatment time from 50 to 10 days and increase the degradation of lignin from 57.8 to 80 % compared with the solid-state fermentation [14], it has two major drawbacks, including large cover area and long fermentation cycle. Therefore, it is essential to develop a new method for CS pretreatment. In the present study, we produced the LiP-containing broth using the method of liquid-state fermentation and used H_2_O_2_-LiP hydrolysis in the pretreatment of CS. Our newly developed method had several advantages, including short pretreatment cycle, small cover area, relatively high efficiency, significantly reduced carbohydrate loss, and no need for special equipment.

## Results and discussion

### Optimization of LiP synthesis conditions

#### One-factor-at-a-time design

It has been proved that *A. oryzae* CGMCC 5992 can secrete LiP, endo-1,4-β-d-glucanase, and proteinase [14]. These enzymes are inducible and involved in the CS degradation. However, no single compound could induce their synthesis at the same time. In contrast, CS contains lignin, cellulose, and hemicellulose, and it can induce the simultaneous synthesis of above-mentioned enzymes. Therefore, CS was selected as the inducer in the entire experiment. Moreover, LiP activity was used as an index to optimize the fermentation condition of *A. oryzae*.

**Fig. 1:**
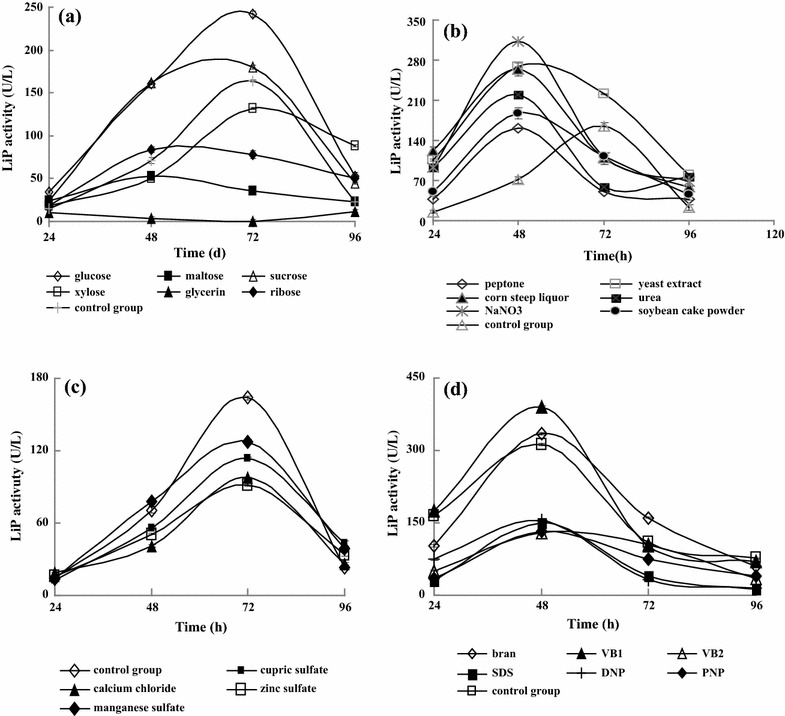
Effects of different factors on LiP activity. **a** carbon sources; **b** nitrogen sources; **c** inorganic salts; **d** other factors

LiP is an inducible enzyme, and small amount of carbon source favors mycelium growth and facilitates LiP synthesis [17, 18]. However, various carbon sources exert significantly different effects on LiP synthesis. Figure 1a shows that the LiP activities produced from all carbon sources reached their maximal values after 2–3 days culture. Compared with the control group without carbon source, the LiP activity was significantly increased due to the addition of glucose and sucrose (*p* < 0.01). Among all tested carbon sources, the LiP activity of the glucose group was the highest (242 ± 3.22 U/L), whereas the lowest LiP activity was detected in the xylose group at 72 h. The same finding has been obtained from the fermentation of *Phanerochaete chrysosporium* for LiP production [19].

For LiP production from different organisms, the effect of nitrogen source shows controversial results from organism to organism [20, 21]. Some strains need excess nitrogen to produce LiP, while LiP from other strains can be induced by nitrogen starvation only. Therefore, nitrogen sources are also regarded as the key factors affecting extracellular LiP production from organisms. As three different types of nitrogen sources, NaNO_3_ is a source of inorganic nitric nitrogen; corn steep liquor is that of organic nitrogen, and its main components include polypeptide and amino acid; yeast extract is also a source of organic nitrogen, containing protein, amino acids, trace vitamin nucleosides, and other nutrients. Therefore, they were selected as nitrogen sources in the present study. Figure 1b shows the optimized medium formulation for LiP production. When NaNO_3_, yeast extract, and corn steep liquor were used as nitrogen sources, the LiP activities were 312 ± 4.85, 268 ± 13.25, and 264 ± 11.26 U/L at 48 h, respectively. It indicated that the time at peak value of LiP activity was shortened from 72 h to 48 h and LiP activities were significantly increased compared with the control group without nitrogen source (164 ± 7.14, *p* < 0.01).

To investigate the effect of inorganic salts on LiP production, CuSO_4_, MnSO_4_, CaCl_2_, and ZnSO_4_ were added to the minimal medium (MM) at a final concentration of 10 mg/L according to our preliminary experiment and the value reported in some literature [22, 23]. Figure 1c shows that the LiP activity was inhibited by these four inorganic salts compared with the control group without inorganic salt. Therefore, no other additional inorganic salts were added to medium.

Wheat bran contains many nutritional factors, including vitamin A (VA), vitamin C (VC), vitamin E (VE), and so on. VB_1_ and VB_2_ are often used as nutritional factors in fermentation. DNP and PNP, both being energy uncouplers, can reduce energy charge regulation by accelerating the NADH hydrolysis and inhibiting ATP synthesis to promote the cellulase synthesis.

As a widely used amphipathic anion surfactant, sodium dodecyl sulfate (SDS) plays various roles upon interaction with proteins, such as protein refolding [24, 25], enzyme activation [26–28], and protein solubilization at the CMC level. It is used more often than any other surfactants as an excellent denaturing or “unfolding” surfactant [29]. Since the addition of SDS to proteins invariably leads to the loss of their biological activities, it is often naively believed that SDS completely unfolds proteins [29–31], a belief perpetuated by SDS-PAGE protocols [32]. In fact, SDS has, for a long time, been shown to induce and stabilize secondary structure, particularly *ɑ*-helices [29, 31]. In the present study, we investigated whether these factors contributed to LiP synthesis. Figure 1d shows that VB_1_ and wheat bran significantly enhanced the LiP activity compared with the control group without other factors (*p* < 0.01). Because wheat bran contains VB_1_ and LiP activity of the wheat bran group was lower than that of the VB_1_ group, VB_1_ was selected in further study.

#### Optimization of LiP synthesis conditions using response surface methodology (RSM)

Based on the results of the one-factor-at-a-time design, we selected glucose, NaNO_3_, yeast extract, corn steep liquor, VB_1_, and wheat bran as the most influential nutrients. Moreover, we further investigated the effects of their interactions on the LiP production by *A. oryzae* CGMCC 5992 liquid-state fermentation using Box–Behnken Design (BBD) of RSM at three levels, resulting in a total of 46 experiments (Additional file 1). Three levels were coded as −1, 0, and +1 [33]. The second-order polynomial equation was based on Eq. (1) as follows:

1 $$Y = \beta_{0} + \sum {\beta_{i} X_{i} + \sum {\beta_{ij} X_{i} X_{j} + \sum {\beta_{ii} X_{i}^{2} } } }$$

where *Y* is the predicted response; *β*_*0*_ is the constant; *β*_*i*_ is the linear coefficient; *β*_*ij*_ is the interaction coefficient; *β*_*ii*_ is the quadratic coefficient; and *X*_*i*_ and *X*_*j*_ are the coded independent variables [21].

Additional file 2 summarizes the results of analysis of variance (ANOVA). The obtained coefficient of determination (*R*^2^) was 0.9066, which was higher than the reported highest *R*^2^ value (0.80) in a well-fitting model [34], indicating that our present model was relatively more reliable in terms of predictability. *F* and *p* values of the model were 12.14 and less than 0.0001, respectively, suggesting that the model fit was significant (*p* < 0.05). The *p* values of “Prob > *F*” were less than 0.05, indicating that the terms were significant. In this case, terms of *X*_2_, *X*_5_, *X*_1_*X*_3_, *X*_1_*X*_4_, *X*_2_*X*_4_, *X*_3_*X*_5_, *X*_1_^2^, *X*_2_^2^, *X*_3_^2^, *X*_4_^2^, and *X*_5_^2^ were significant model terms. Therefore, the model contained two linear (*X*_2_ and *X*_5_), five quadratic (*X*_1_, *X*_2_, *X*_3_, *X*_4_, and *X*_5_), and five interaction (*X*_1_*X*_3_, *X*_1_*X*_4_, *X*_2_*X*_4_, *X*_2_*X*_5_, and X_3_X_5_) terms plus one block term according to the Design-Expert version (8.0.4). Since the model showed insignificant lack of fit (*p* = 0.1334), the response could be sufficiently explained by the regression equation as follows:

2 $$\begin{aligned} Y = 642 + 40.31X_{2} + 35.44X_{5} - 69.25X_{1} X_{3} + 155X_{1} X_{4} - 100.75X_{2} X_{4} + 112X_{2} X_{5} \hfill \\ + 104.5X_{3} X_{5} - 277.75X_{1}^{2} - 163.5X_{2}^{2} - 95.25X_{3}^{2} - 83.58X_{4}^{2} - 85.5X_{5}^{2} \hfill \\ \end{aligned}$$

where *Y* is LiP activity; *X*_1_, *X*_2_, *X*_3_, *X*_4_, and *X*_5_ denote glucose, sodium nitrate, corn steep liquor, yeast extract, and VB_1_, respectively.

**Fig. 2:**
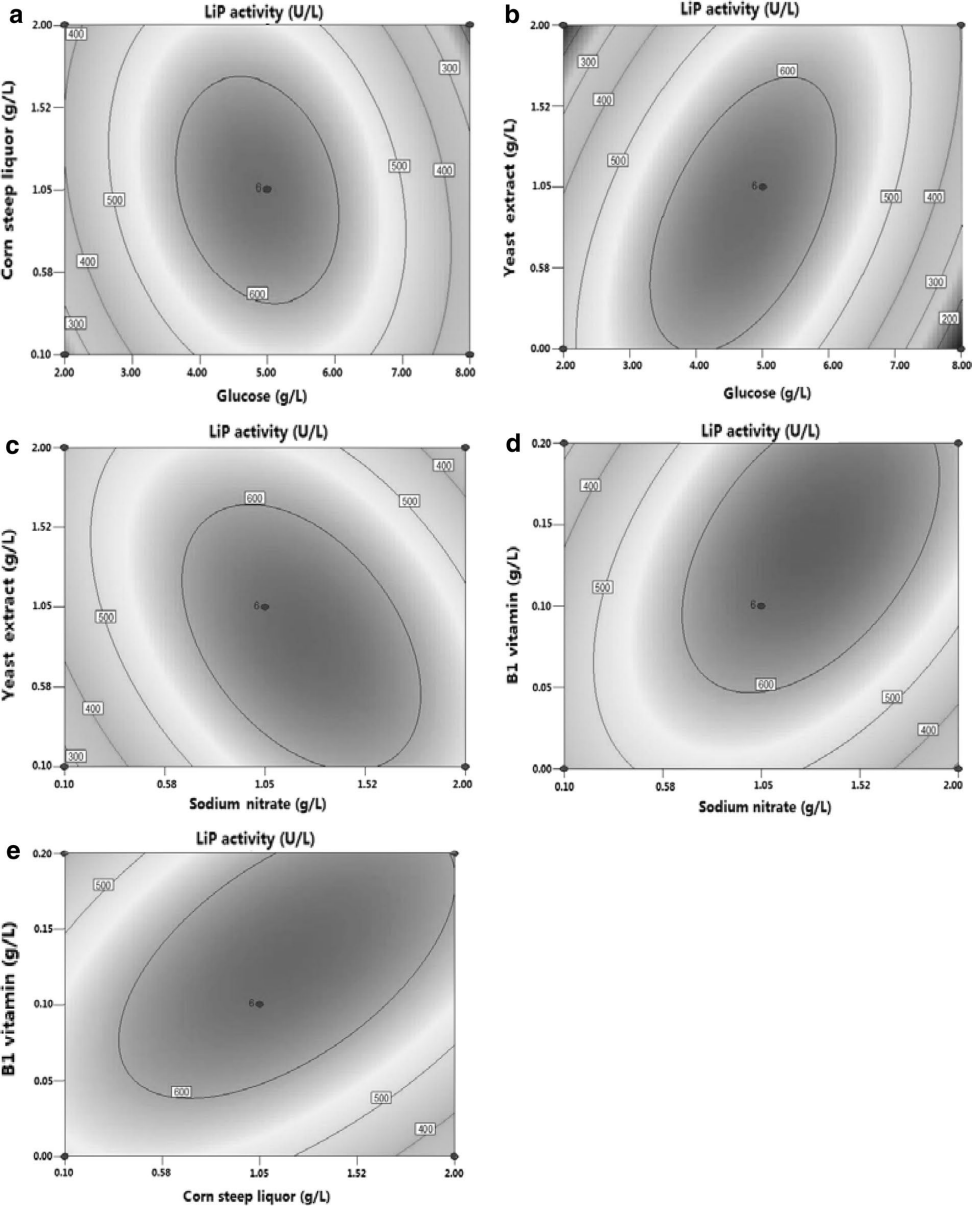
Contour lines of interactions between different factors on LiP production during the fermentation process of *A. oryzae* CGMCC5992. **a** Glucose and corn steep liquor; **b** Yeast extract and glucose; **c** Yeast extract and NaNO_3_; **d** VB_1_ and NaNO_3_; **e** Corn steep liquor and VB_1_

The contour plots are commonly used to study the interaction among various factors and to find out the optimal value of each factor for maximal production [35, 36]. Figure 2 exhibits 2D contour plots with the effects of corn steep liquor and glucose; yeast extract and glucose; NaNO_3_ and yeast extract; VB_1_ and NaNO_3_; and VB_1_ and yeast extract on the response (LiP activity). In addition, we performed BBD analysis to investigate the combined effect of corn steep liquor and glucose on LiP activity in broth. Figure 2a shows that when the concentrations of glucose and corn steep liquor were increased from 2 to 5.0 g/L and from 0.1 to 1 g/L, respectively, the LiP activity was rapidly increased. However, when the concentrations of glucose and corn steep liquor were further increased from 6 to 8 g/L and from 1.2 to 2 g/L, respectively, the LiP activity was sharply decreased. The maximum LiP activity was obtained when the concentrations of glucose and corn steep liquor were 4.5–5.5 and 0.3–1.05 g/L, respectively. The relationship among glucose, yeast extract, and LiP activity is illustrated as the contour line in Fig. 2b, showing that the LiP activity was slowly increased with the increasing yeast extract concentration from 0.1 to 1.1 g/L, and it reached the maximum at 1.1 g/L. Figure 2b reveals that the maximum LiP activity was obtained under a particular range of glucose (4.8–5.5 g/L) and yeast extract (1.05–1.25 g/L). Figure 2c shows the effect of interaction between NaNO_3_ and yeast extract on LiP activity, clearly revealing that the LiP activity varied with the concentrations of NaNO_3_ and yeast extract (0.1–2 g/L). Moreover, three nitrogen sources showed similar effects on LiP activity (Fig. 2a–c). The LiP activity reached its maximum when NaNO_3_ and yeast extract were added within a certain range (NaNO_3_ at 0.8–1.2 g/L and yeast extract at 1–1.2 mg/L).

Figure 2d, e reveals that interactions between VB1 and NaNO_3_ or between VB_1_ and corn steep liquor exerted the same effect on LiP activity. When the concentration of NaNO_3_ or yeast extract was increased from 0.1 to 1.05 g/L and the concentration of VB_1_ was increased from 0 to 0.10 g/L, the LiP activity was slowly increased. However, when the concentration of NaNO_3_ or yeast extract exceeded 1.2 g/L and the concentration of VB_1_ was more than 0.15 g/L, the LiP activity was slowly decreased with their increasing concentrations. The highest LiP activity was obtained under a particular range of VB_1_ (0.09–0.12 g/L) and NaNO_3_ or yeast extract (0.9–1.2 g/L).

The interactions between glucose and yeast extract, and between VB_1_ and NaNO_3_ or yeast extract were associated with a positive regression coefficient in the equation, indicating that that the increase of LiP activity was favored by an increase in these factors up to certain values, while the LiP activity would decline beyond these values.

In conclusion, One-factor-at-a-time design and RSM were applied to optimize the medium components for LiP synthesis. The determined optimal medium contained CS of 30 g/L, glucose of 4.6 g/L, sodium nitrate of 1.2 g/L, corn steep liquor of 1 g/L, yeast extract of 1.2 g/L, and vitamin B_1_ of 0.15 g/L. Under the optimal conditions, the experimentally determined LiP activity reached its maximum of 652.34 U/L, which was much higher than reported values in other studies [37–41].

### Optimization of lignin degradation conditions using H_2_O_2_ hydrolysis catalyzed by LiP

Although it is believed that lignin is the key obstacle to degrade cellulose in the stalk, sugar yield of stalk is not proportional to the lignin content in degradation [42]. Therefore, the sugar yield rather than removal of lignin is more suitable as an index to reflect the degree of degradation.

#### One-factor-at-a-time design

There are ten key factors affecting the reaction rate, including the fermentation broth amount (FBA); concentration; amount and flow rate of H_2_O_2_; water/material ratio; pH and temperature in reaction mixture; and reaction time, as well as temperature and time of pretreatment. The solid substrate has a strong ability to buffer changes in pH. Adjustment of pH in reaction solution containing solid substrate requires much more acids and alkalis, leading to a large amount of salt residues in sugar solution for the next step of cellulose hydrolysis. These residues are unfavorable to alcohol fermentation. Considering the subsequent alcohol fermentation, the hydrolysis was carried out under conditions of natural pH.

**Fig. 3:**
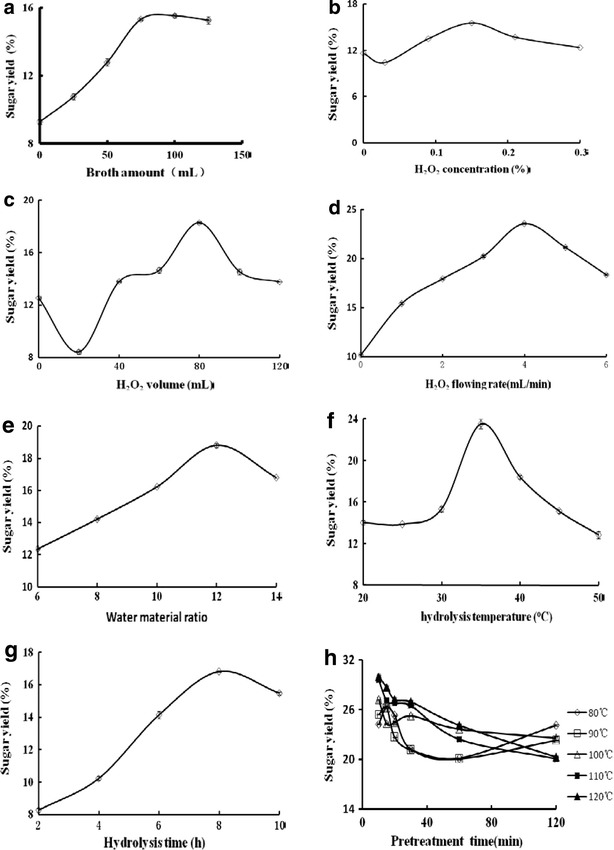
The effects of various factors on CS hydrolysis by LiP-H_2_O_2_. **a** Broth volume including 631U/L LiP, 76 U/L MnP; **b** H_2_O_2_ concentration; **c** H_2_O_2_ volume; **d** H_2_O_2_ flow rate; **e** water/material ratio; **f** hydrolysis temperature; **g** Hydrolysis time; **h** pretreatment temperature and time

FBA is the key factor to control speed of CS hydrolysis. In the present study, enzyme amount meant the FBA of *A. oryzae* CGMCC 5992. We then compared the effect of different FBAs on the sugar yield using following parameters: CS of 20 g, H_2_O of 200 mL, 0.15 % H_2_O_2_ solution of 100 mL, H_2_O_2_ flow rate of 0.4 mL/min, and stir speed of 100 rpm. When FBA was extremely low, the CS hydrolysis rate was proportional to the FBA. Figure 3a shows that the sugar yield was linearly increased with the increasing FBA when the FBA was less than 75 mL. However, when the FBA was greater than 75 mL, the sugar yield was not increased with the -increasing FBA (Fig. 3a). Therefore, FBA of 75 mL was a suitable volume for the reaction.

As another substrate for LiP to catalyze the lignin hydrolysis, H_2_O_2_ is closely related with the removal rate of lignin. High concentration of H_2_O_2_ can accelerate the delignification, leading to the LiP inactivation. To protect enzymes in the reaction solution from inactivation, H_2_O_2_ solution was gradually added to the reaction mixture by continuous flow. H_2_O_2_ concentration in reaction mixture was dependent on flow rate, volume, and concentration of H_2_O_2_ during the process of continuous flow. Therefore, we also compared the effect of different flow rates, volumes, and concentrations of H_2_O_2_ on the sugar yield. The selected ranges of the concentration, volume, and flow rate of H_2_O_2_ were 0–3 %, 0–120 mL, and 0–6 mL/min, respectively. Figure 3b–d shows that when the concentration, volume, and flow rate of H_2_O_2_ were less than 1.5 %, 75 mL, and 4 mL/min, respectively, the sugar yield was positively correlated with those factors. However, when they were greater than those above-mentioned values, the sugar yield was negatively correlated with them.

A high water/material ratio is beneficial to the lignin hydrolysis. However, it is cost-inefficient, leading to a low sugar concentration in the hydrolysis solution. Therefore, we further optimized the water/material ratio in the reaction. Figure 3e clearly exhibits that the optimal water/material ratio was 12:1. Sugar yield was increased with the -increasing water/material ratio, and it reached its maximum at a water/material ratio of 12:1.

In order to explore the optimal temperature of hydrolysis, the hydrolysis reaction was carried out at a series of temperatures (20, 25, 30, 35, 40, 45, and 50 °C). Figure 3f shows that the profile curve for hydrolysis temperature was in accordance with the bell-shaped curve of the general enzyme reaction. The highest sugar yield (23.2 ± 0.46 %) was obtained at 35 °C.

Reaction time is a key factor for CS hydrolysis. The hydrolysis is not fully completed in a shorter duration, whereas the longer reaction time leads to several by-products and low sugar yield. In the present study, we investigated the effect of different reaction time on sugar yield. Figure 3g shows that the sugar yield reached its maximum at 8 h, therefore reaction time of 8 h was selected as the optimum.

CS can be fully swollen with pretreatment at higher temperature, leading to increased contact area of each component in CS and enzymes in broth and enhanced sugar yield. Temperature and time of pretreatment are the two key factors affecting the pretreatment. Therefore, we compared the effect of different times and temperatures of pretreatment on sugar yield. Figure 3h clearly shows that pretreatment at a higher temperature significantly improved the sugar yield, and the higher the pretreatment temperature, the shorter the pretreatment time required. The highest sugar yield (45 ± 0.42 %) was obtained at the pretreatment temperature of 110–120 °C and the pretreatment time of 10–20 min.

#### Optimization by RSM

Based on the results of the one-factor-at-a-time design, in addition to reaction time, a total of eight factors were divided into two groups and further optimized by two BBDs of RSM at three levels, resulting in a total of 29 experiments. Because the enzyme activity was closely related with H_2_O_2_ concentration in the reaction solution, the first BBD of RSM included the FBA as well as the concentration, flow rate, and amount of H_2_O_2_ (Additional file 3), while the other BBD of RSM included the water/material ratio and reaction temperature as well as the temperature and time of CS pretreatment (Additional file 4). Three levels were coded as −1, 0, and +1, respectively [43]. The second-order polynomial equation was based on Eq. (1).

Additional files 5 and 6 summarize the ANOVA of the response variables for the quadratic polynomial model. The F-values of the two models were 16.12 and 9.913, respectively, and the *p* values of the two models were all less than 0.0001. FBA in the first RSM with an F-value of 120.8 and the pretreatment time in the other RSM with an *F* value of 41.71 were the most significant factors in the two RSM analyses, respectively. The *p*-value of “Prob > *F*” in the model terms was less than 0.0001, indicating that the terms were extremely significant. Therefore, *X*_3_, *X*_4_, *X*_3_*X*_4_, *X*_1_^2^, *X*_2_^2^, *X*_3_^2^, and *X*_4_^2^ were significant model terms in the first RSM. The p-values of “lack of fit” in the two models were 0.2901 and 0.1237, respectively, implying that the “lack of fit” was not significant. These results suggested that the two models were statistically meaningful and could be effectively used to discuss the relationship between the independent variables and response variables [44]. The *R*^2^ values of the two models were 0.9416 and 0.9084, respectively, revealing that the high coefficient of determination suggested a strong correlation between the observed values and the predicted values in the two models [45]. The quadratic polynomial equations of two RSM analyses as obtained by multiple regression analysis are shown in Eqs. (3) and (4).

3 $$\begin{aligned} Y = 16.92 + 3.89X_{1} + 0.77X_{3} + 2.34X_{4} - 1.5X_{1} X{}_{2} + 1.5X{}_{1}X{}_{3} \hfill \\ + 2.56X_{2} X_{3} + 0.14X_{1}^{2} - X_{2}^{2} - 0.85X_{3}^{2} + 1.67X_{4}^{2} \hfill \\ \end{aligned}$$

where *Y* is sugar yield; *X*_1_, *X*_2_, *X*_3_, and *X*_4_ denote FBA, H_2_O_2_ concentration, H_2_O_2_ flow rate, and H_2_O_2_ volume, respectively.

4 $$\begin{aligned} Y = 28.68 - 0.29X_{1} - 0.40X_{2} + 1.79X{}_{3} + 2.81X_{4} - 2.27X_{3} X_{4} \hfill \\ - 1.9X_{1}^{2} - 3.05X_{2}^{2} - 2.02X_{3}^{2} - 3.93X_{4}^{2} \hfill \\ \end{aligned}$$

where *Y* is sugar yield; *X*_1_, *X*_2_, *X*_3_, and *X*_4_ denote water/material ratio, hydrolysis temperature or reaction temperature, pretreatment temperature, and pretreatment time, respectively.

**Fig. 4:**
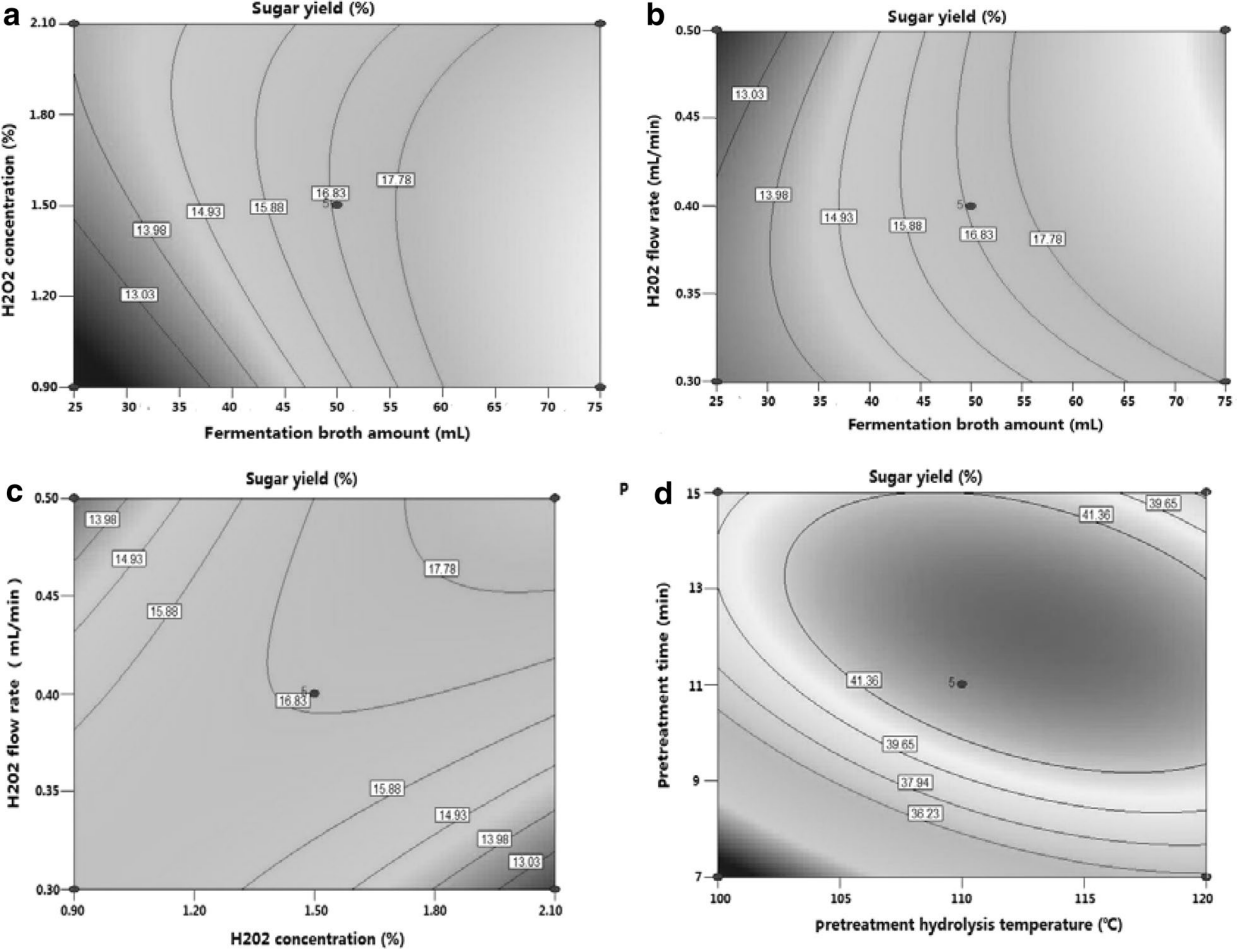
Contour line of interaction between different factors during the process of hydrolysis. **a** Fermentation broth amount and H_2_O_2_ concentration; **b** fermentation broth amount and H_2_O_2_ flow rate; **c** H_2_O_2_ concentration and H_2_O_2_ flow rate; **d** pretreatment temperature and pretreatment time

To investigate the combined effects of FBA and H_2_O_2_ concentration; FBA and H_2_O_2_ flow rate; H_2_O_2_ concentration and H_2_O_2_ flow rate; or pretreatment temperature and pretreatment time on sugar yield, we performed the BBD analysis, and the contour lines of their interactions were accordingly generated (Fig. 4). Figure 4a–c clearly shows that the sugar yield was increased with the increasing FBA from 25 to 75 mL. When the FBA was fixed, the sugar yield was positively correlated with the H_2_O_2_ concentration and flow rate within a certain scope (H_2_O_2_ concentration of 0.9–1.3 %; H_2_O_2_ flow rate of 0.3–0.38 mL/min).

Our results of two RSM analyses proved again that the pretreatment of CS at high temperature was necessary and important for the sugar yield. The CS in all experiments of the first RSM was not pretreated at high temperature, leading to a low sugar yield in the first RSM compared with the second RSM. The contour lines of pretreatment temperature and pretreatment time (Fig. 4d) showed that the sugar yield rapidly reached its maximum when the pretreatment temperature and time were increased from 100 to 114 °C and from 0 to 12 min, respectively. However, the sugar yield was slowly decreased when the pretreatment temperature and time exceeded 114 °C and 12 min, respectively. The interaction of pretreatment temperature and time showed a negative regression coefficient in the equation, indicating that the sugar yield was negatively correlated with their interactions up to certain values.

Based on the above-mentioned results of one-factor-at-a-time design and two RSM analyses, the optimal conditions for CS hydrolysis by H_2_O_2_-LiP catalysis included CS of 20 g, FBA of 50 mL, H_2_O_2_ concentration of 5 %, H_2_O_2_ volume of 80 mL, H_2_O_2_ flow rate of 0.4 mL/min, water/material ratio of 10:1, hydrolysis temperature of 39 °C, and hydrolysis time of 8 h. Sugar yield could be increased when CS was pretreated at 113 °C for 11 min. The predicted sugar yield under the optimal conditions was 45.56 %, and the actual experimentally determined value was 46.28 %, which was 4.07 times higher compared with the sugar yield (11.19 %) without H_2_O_2_-LiP catalysis. In addition, our sugar yield was also close to the reported values in previous studies [46–48].

### Scanning electron microscopy (SEM)

**Fig. 5:**
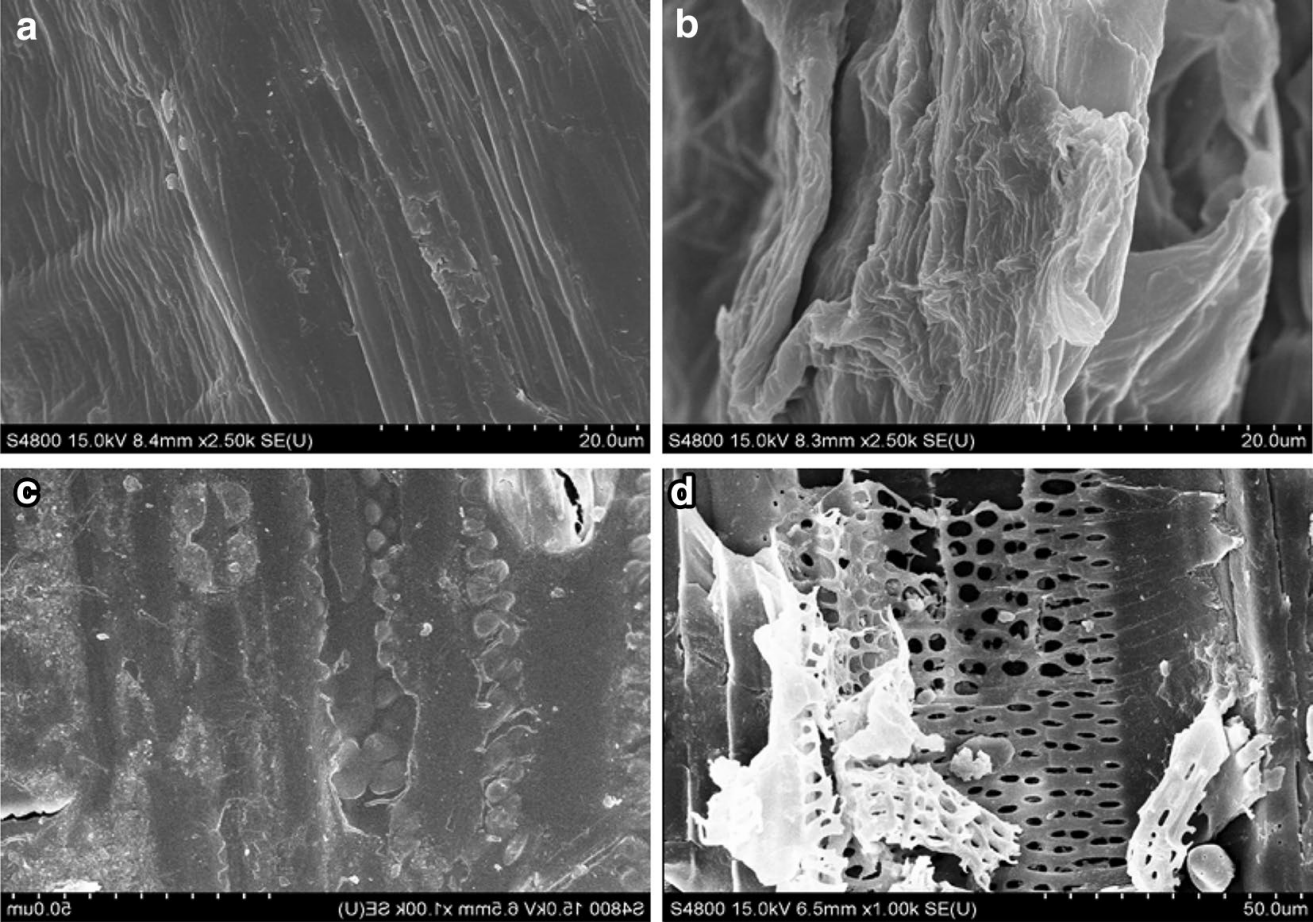
SEM images for CS in different stages. **a** Raw material (×2000); **b** after fermentation (×2000); **c** pretreated CS (×1000); **d** residue after cellulase hydrolysis (×1000)

SEM was used to examine micro- and ultrastructural changes of CS before and after the fermentation as well as in the process of pretreatment, including after the H_2_O_2_-LiP hydrolysis in the fermentation broth and after the cellulose hydrolysis (Fig. 5). Raw CS has a tight structure with vascular bundles, in which lignin surrounds cellulose and hemicellulose (Fig. 5a) [49]. After the fermentation, the CS showed a loose structure, and part of vascular bundles was degraded (Fig. 5b). Because many literature studies have reported that LiP of extracellular microorganism can catalyze the reaction that microorganism-synthesized H_2_O_2_ oxidizes and degrades lignin, and the strain can synthesize LiP in the process of fermentation, it is reasonable to deduce that the loose structure of CS and partly degraded bundles were caused by the swelling effect of CS soaked in water for 2 days and high-temperature effect during the process of sterilization and oxidative degradation of LiP-H_2_O_2_. Figure 5c shows that the holes and strips remained around the vascular bundles after the lignin was hydrolyzed by H_2_O_2_-LiP in the fermentation broth. Furthermore, the rudimental lignin sheath with these holes and strips became more evident after the cellulose was hydrolyzed (Fig. 5d).

## Conclusions

In the present study, H_2_O_2_-LiP pretreatment of CS was successfully developed in order to convert CS into sugar solution. The medium for *A. oryzae* CGMCC 5992 to prepare fermentation broth was optimized with univariate analysis and BBD of RSM. The optimal medium contained 30 g/L CS, 4.6 g/L glucose, 1.2 g/L sodium nitrate, 1 g/L corn steep liquor, 1.2 g/L yeast extract, and 0.15 g/L vitamin B_1_. Under these conditions, the LiP activity reached its maximum (652.34 U/L). The optimal conditions for CS hydrolysis by H_2_O_2_-LiP catalysis was obtained by univariate analysis and two BBDs of RSM. These optimized conditions included CS of 20 g, FBA of 50 mL, H_2_O_2_ concentration of 5 %, H_2_O_2_ volume of 80 mL, H_2_O_2_ flow rate of 0.4 mL/min, water/material ratio of 10:1, hydrolysis temperature of 39 °C, and hydrolysis time of 8 h. Under these conditions, the highest sugar yield was 46.28 %, which was 4.07 times higher compared with unpretreated CS. Taken together, our findings indicated that pretreatment of CS by H_2_O_2_-LiP possessed great advantages in bio-ethanol production.

## Methods

### Materials

CS was purchased from a local farm and ground to fine powders through a 0.25-mm sieve. The main components (dry weight basis) of the ground corn stover consisted of 26.24 % hemicellulose, 32.12 % cellulose, and 15.42 % lignin. All chemicals were of analytic or reagent grade.

### Microorganism

In the present study, *A. oryzae* CGMCC5992 was isolated from the sludge of the Yudai River in Jiangsu University and stored in China General Microbiological Culture Collection Center (CGMCC, China). The strain was cultured on the potato dextrose agar (PDA) slants at 28 °C for 4 days, then stored at 4 °C and passaged every 7–9 weeks.

### LiP preparation

A total of 1 × 10^6^ spores from the *A. oryzae* CGMCC 5992 strain were aseptically inoculated into a 250-mL Erlenmeyer flask containing 100 mL potato dextrose (PD) medium and then incubated at 35 °C for 24 h at 125 rpm in a rotary shaking incubator to produce a mass of pellets. This pellet culture was used as seed in the liquid-state fermentation.

Briefly, 10 mL seed culture was aseptically inoculated into 250-mL Erlenmeyer flask containing 100 mL minimal medium (MM) (pH 6.8–7.0), which was composed of 5 g/L glucose, 30 g/L CS powder, 3 g/L (NH_4_)_2_SO_4_, 1 g/L KH_2_PO_4_, 0.5 g/L NaCl, 0.5 g/L MgSO_4_·7H_2_O and 0.01 g/L FeSO_4_. After inoculation, the flask was incubated at 35 °C for 120 h at 125 rpm in a rotary shaking incubator. During the culturing process, 2 mL sample was collected to determine enzyme activity at an interval of 24 h. After the sample was centrifuged, the supernatant was collected to determine the LiP activity.

### Hydrolysis of CS catalyzed by *A. oryzae* CGMCC 5992 broth

In brief, 20 g CS, 250 mL H_2_O, and 75 mL FBA were mixed in a 500-mL three-necked round-bottom flask, and the mixture was stirred at 100 rpm and preheated to 35 °C in the water bath. Subsequently, 100 mL H_2_O_2_ (1.5 %) was added to the mixture at the flow rate of 0.5 mL/min. After hydrolysis for 8 h, the mixture was filtered. The residue was washed with distilled water and then dried to a constant weight at 105 °C to determine the sugar yield. In addition, the filtrate and washing solution were also combined to determine the sugar content.

### Analysis

#### Determination of LiP and MnP activities

LiP activity was spectrophotometrically determined according to a previously described method [50]. The reaction solution included 1.5 mL of 10 mM veratryl alcohol, 50 μL enzyme sample, and 0.25 M sodium tartrate buffer (pH 2.5). The reaction was initiated by adding H_2_O_2_ (5 mM). One unit (U) of LiP activity was defined as the amount of enzyme required to oxidize 1 μmol veratryl alcohol to veratryl aldehyde in 1 min at 30 °C. The activity of MnP was measured by UV–Vis spectroscopy (UV-1801, Lite Electronic Science and Technology Co. Ltd., China) at 240 nm using Mn(II) as a substrate according to Kuwahara et al. [51]. One unit of MnP was defined as the amount of enzyme required to oxidize 1 μmol of substrate in 1 min at 25 °C.

#### Determination of sugar yield

Cellulase solution was a commercial *Trichoderma reesei* cellulase purchased from Guangzhou Global Green Tech. Ltd., China. The activities of carboxymethyl-cellulase, filter paper enzyme, β-glucosidase, and hemicellulase were 5.97 × 10^4^ U/mL, 821 FPU/mL, 10.1 U/mL, and 284 U/mL, respectively. The hydrolysis experiment was conducted in 100-mL Erlenmeyer flasks consisting of 1.0 g residue from CS after the H_2_O_2_-LiP hydrolysis, 0.1 mL liquid cellulase, and 20 mL distilled water at pH 6.0. The hydrolysis mixture was incubated at 50 °C in an orbital shaker at 110 rpm for 10 h. Subsequently, the hydrolysis mixture was filtered, and the residue was washed with distilled water. The filtrate and washing solution were combined to determine the sugar content.

Sugar content was determined according to the 3.5-dinitrosalicylic acid method [52]. The sugar yield was calculated using an equation as follows:

$$Y = \frac{{C_{1} \times V_{1} + C_{2} \times V_{2} \times W_{1} }}{W} \times 100\,\%$$

where *Y* represents the sugar yield; *C*_1_ and *V*_1_ are the sugar content and the combined volume of filtrate and washing solution after H_2_O_2_-LiP hydrolysis, respectively; *C*_2_ and *V*_2_ are the sugar content and the combined volume of filtrate and washing solution after cellulase hydrolysis, respectively; *W*_1_ is the weight of residue after H_2_O_2_ hydrolysis; *W* is the weight of initial CS.

#### Chemical analysis of CS

After dried to a constant weight, 10 g of untreated CS was milled by passing through an 80-mesh sieve. The cellulose, hemicellulose, and lignin contents of untreated CS were determined following the National Renewable Energy Laboratory-Laboratory Analytical Procedures for standard biomass analysis (NREL-LAP) by two-step acid hydrolysis [53]. Lignin is the sum of acid-soluble and acid-insoluble lignin. The acid-insoluble lignin was measured by gravimetric analysis developed by Han and Rowell [54], and the acid-soluble lignin was measured by UV–Vis spectroscopy (UV-1801, Lite Electronic Science & Technology Co. Ltd., China). All analyses were performed at least in triplicate, and the results were presented as the means.

### Data analysis

All the tests were repeated at least three times, and the results were presented as mean ± standard error.

### SEM observation of untreated and pretreated CS

The morphological properties of raw CS or CS after fermentation, H_2_O_2_-LiP hydrolysis and cellulose hydrolysis were examined by a Hitachi S-4800 microscope (Japan). All samples were dried at 105 °C to constant weight and then sputter-coated with Au–Pd prior to observation.

### Experimental designs

#### Optimization of nutritional constituents for LiP production by A. oryzae CGMCC 5992

*One*-*factor*-*at*-*a*-*time test* Various carbon sources including glucose, maltose, sucrose, xylose, ribose, and glycerin at a concentration of 5 g/L; various nitrogen sources including peptone, yeast extract, corn steep liquor, NaNO_3_, urea and soybean powder at a concentration of 3.0 g/L; various minerals including manganese sulfate, cupric sulfate, zinc sulfate, and calcium chloride at a concentration of 0.5 g/L; and various other impact factors including bran, vitamin B_1_ (VB_1_), vitamin B6 (VB_6_), 2,4-dinitrophenol (DNP), *p*-nitrophenol (PNP), and sodium dodecyl sulfate (SDS) at a concentration of 0.05 g/L were used to substitute the corresponding components in MM or supplement into MM in order to investigate the effect of different factors on LiP activity in the broth.

*Response surface designs* According to the results of one-factor-at-a-time test, five independent variables, including glucose (*X*_1_), sodium nitrate (*X*_2_), corn steep liquor (*X*_3_), yeast extract (*X*_4_), and VB_1_ (*X*_5_), were selected to further optimize response surface experiment using the LiP activity as an index. Table 1 lists the detailed information of the range and levels of the five factors in the optimization study. A total of 46 runs were performed to optimize the parameters according to the design. The response value in each run was expressed as the average of triplicates. The experimental design for RSM was developed using Design-Expert Software (version 5.0.9; Stat-Ease Corporation, USA). The RSM experiments were conducted at 35 °C, 125 rpm for 120 h in a rotary shaking incubator.

**Table 1:** Experimental range and levels of the independent variables in terms of actual and coded factors in the optimization of components for LiP synthesis

Independent variable	Symbol	Level (g/L)		
		−1	0	1
Glucose	*X*_1_	2	5	8
Sodium nitrate	*X*_2_	0.1	1.1	2
Corn steep liquor	*X*_3_	0.1	1.1	2
Yeast extract	*X*_4_	0.1	1.1	2
VB_1_	*X*_5_	0	0.01	0.02

#### Condition optimization of the LiP-H_2_O_2_ delignification

*One*-*factor*-*at*-*a*-*time test* In the present study, we investigated the effect of nine factors; including FBA; H_2_O_2_ concentration; flow rate; H_2_O_2_ volume; water/material ratio; temperature and time of hydrolysis; and temperature and time of pretreatment, on the sugar yield using 20 g CS. The FBA used in the experiment contained 631U/L LiP and 76 U/L MnP. No laccase activity of FBA has been assayed.

*Response surface designs* Based on the results of one-factor-at-a-time test, eight factors significantly affecting the sugar yield were divided into two groups to perform two RSM analyses with four factors and at three levels. The four independent parameters in the first RSM were FBA (*X*_1_), H_2_O_2_ concentration (*X*_2_), H_2_O_2_ flow rate (*X*_3_), and H_2_O_2_ volume (*X*_4_). Table 2 lists their range and levels of the four factors in the optimization study. The RSM experiments were conducted under conditions as follows: unpretreated CS of 20 g, water/material ratio of 10:1, hydrolysis temperature at 35 °C, and 125 rpm for 8 h. The water/material ratio (*X*_1_), hydrolysis temperature (*X*_2_), pretreatment temperature (*X*_3_), and pretreatment time (*X*_4_) were the four independent factors of another RSM. Table 3 shows their ranges and levels in the optimization study. Sugar yield was used as the index. The response value in each run was expressed as the average of triplicates. The RSM experiments were conducted under conditions as follows: CS of 20 g, FBA 50 mL, H_2_O_2_ concentration of 1.5 %, H_2_O_2_ flow rate of 0.4 mL/min, and H_2_O_2_ volume of 100 mL. The experimental design for RSM was developed using Design-Expert Software (version 5.0.9; Stat-Ease Corporation, USA) [55].

**Table 2:** Experimental range and levels of the independent variables in terms of actual and coded factors in the optimization of enzyme amount and H_2_O_2_ flow rate

Independent variable	Symbol	Level		
		−1	0	1
Enzyme volume	*X*_1_	25	50	75
H_2_O_2_ concentration (%)	*X*_2_	0.9	1.5	2.1
H_2_O_2_ flow rate	*X*_3_	0.3	0.4	0.5
H_2_O_2_ volume	*X*_4_	60	80	100

**Table 3:** Experimental range and levels of the independent variables in terms of actual and coded factors in the optimization of conditions of hydrolysis reaction

Independent variable	Symbol	Level		
		−1	0	1
Water material ratio	*X*_1_	8	10	12
Hydrolysis temperature	*X*_2_	35	40	45
Pretreatment temperature (°C)	*X*_3_	100	110	120
Pretreatment time	*X*_4_	5	10	15

## Additional files

10.1186/s13068-015-0362-4 In the Supplemental Material Section Box–Behnken design and the result in the optimization of components for LiP synthesis are presented.
10.1186/s13068-015-0362-4 In the Supplemental Material Section results of regression analysis of quadratic response surface model fitting in the optimization of components for LiP synthesis are presented.
10.1186/s13068-015-0362-4 In the Supplemental Material Section Box–Behnken design and the result in the optimization of fermentation broth amount H_2_O_2_ concentration, H_2_O_2_ amount and H_2_O_2_ flow rate are presented.
10.1186/s13068-015-0362-4 In the Supplemental Material Section Box–Behnken design and the result in the optimization of the conditions of hydrolysis reaction are presented.
10.1186/s13068-015-0362-4 In the Supplemental Material Section results of regression analysis for quadratic response surface model fitting (ANOVA) in the optimization of enzyme amount and H_2_O_2_ flow rate are presented.
10.1186/s13068-015-0362-4 In the Supplemental Material Section the results of regression analysis for quadratic response surface model fitting (ANOVA) in the optimization of the conditions of hydrolysis reaction are present.

## Abbreviations

CS: corn stalk
LiP: lignin peroxidase
MnP: manganese peroxidase
Lac: laccase
RSM: response surface methodology
BBD: Box–Behnken design
ANOVA: analysis of variance
SEM: scanning electron microscopy
FBA: fermentation broth amount
PDA: potato dextrose agar
PD: potato dextrose
MM: minimal medium
VB_1_: vitamin B_1_
VB_6_: vitamin B_6_
DNP: 2,4-dinitrophenol
PNP: p-nitrophenol
SDS: sodium dodecyl sulfate
