# Supplementary material for: Lignin degradation in corn stalk by combined method of H2O2 hydrolysis and Aspergillus oryzae CGMCC5992 liquid-state fermentation
Source: Biotechnol Biofuels. 2015 Nov 19;8:183. doi: 10.1186/s13068-015-0362-4 (PMC4653895; doi:10.1186/s13068-015-0362-4)
Supplement: Supplementary file 1 — 10.1186/s13068-015-0362-4 In the Supplemental Material Section Box–Behnken design and the result in the optimization of components for LiP synthesis are presented. [file 13068_2015_362_MOESM1_ESM.docx]

**Zhang et al. Additional file Table 1**: **In the Supplementa Material Section Box-Behnken design and the result in the optimization of components for LiP synthesis are presented.**

| **Run** | **X_1_** | **X_2_** | **X_3_** | **X_4_** | **X_5_** | **LiP activity (U/L)** |
| --- | --- | --- | --- | --- | --- | --- |
| 1 | -1 | 0 | 0 | -1 | 0 | 480 |
| 2 | 1 | 0 | -1 | 0 | 0 | 361 |
| 3 | 0 | -1 | 0 | 0 | -1 | 472 |
| 4 | 0 | 1 | 0 | 1 | 0 | 340 |
| 5 | 0 | 0 | -1 | 0 | -1 | 395 |
| 6 | -1 | 0 | 0 | 1 | 0 | 125 |
| 7 | 0 | 0 | 0 | 1 | -1 | 536 |
| 8 | -1 | 0 | -1 | 0 | 0 | 282 |
| 9 | 0 | -1 | 1 | 0 | 0 | 294 |
| 10 | 1 | 0 | 0 | 0 | 1 | 302 |
| 11 | 0 | -1 | -1 | 0 | 0 | 317 |
| 12 | -1 | 0 | 0 | 0 | -1 | 358 |
| 13 | 0 | 0 | 0 | 0 | 0 | 654 |
| 14 | -1 | 0 | 0 | 0 | 1 | 342 |
| 15 | 1 | 0 | 0 | -1 | 0 | 124 |
| 16 | 0 | -1 | 0 | -1 | 0 | 202 |
| 17 | 1 | 1 | 0 | 0 | 0 | 245 |
| 18 | 0 | 0 | 0 | 1 | 1 | 364 |
| 19 | 0 | 0 | 1 | 0 | -1 | 249 |
| 20 | 0 | 1 | 1 | 0 | 0 | 435 |
| 21 | 0 | 1 | 0 | 0 | -1 | 312 |
| 22 | 0 | -1 | 0 | 1 | 0 | 453 |
| 23 | 0 | 0 | 1 | -1 | 0 | 483 |
| 24 | 0 | 0 | -1 | 1 | 0 | 402 |
| 25 | 0 | 0 | 1 | 0 | 1 | 642 |
| 26 | 1 | 0 | 1 | 0 | 0 | 156 |
| 27 | -1 | 1 | 0 | 0 | 0 | 136 |
| 28 | 0 | 1 | 0 | -1 | 0 | 492 |
| 29 | 0 | 0 | 0 | 0 | 0 | 684 |
| 30 | 1 | 0 | 0 | 0 | -1 | 135 |
| 31 | 0 | 0 | 0 | -1 | -1 | 474 |
| 32 | -1 | -1 | 0 | 0 | 0 | 209 |
| 33 | 0 | 1 | 0 | 0 | 1 | 641 |
| 34 | 0 | 0 | 0 | 0 | 0 | 678 |
| 35 | -1 | 0 | 1 | 0 | 0 | 354 |
| 36 | 1 | -1 | 0 | 0 | 0 | 124 |
| 37 | 0 | 0 | -1 | -1 | 0 | 592 |
| 38 | 0 | 0 | 0 | 0 | 0 | 662 |
| 39 | 0 | 0 | 0 | -1 | 1 | 484 |
| 40 | 0 | -1 | 0 | 0 | 1 | 353 |
| 41 | 0 | 0 | 1 | 1 | 0 | 512 |
| 42 | 1 | 0 | 0 | 1 | 0 | 389 |
| 43 | 0 | 1 | -1 | 0 | 0 | 468 |
| 44 | 0 | 0 | -1 | 0 | 1 | 370 |
| 45 | 0 | 0 | 0 | 0 | 0 | 586 |
| 46 | 0 | 0 | 0 | 0 | 0 | 590 |

Where X_1_ is glusose; X_2_ is sodium nitrate; X_3_ is corn steep liquor; X_4_ is yeast extract; X5 is VB_1_.
