# Supplementary material for: Lignin degradation in corn stalk by combined method of H2O2 hydrolysis and Aspergillus oryzae CGMCC5992 liquid-state fermentation
Source: Biotechnol Biofuels. 2015 Nov 19;8:183. doi: 10.1186/s13068-015-0362-4 (PMC4653895; doi:10.1186/s13068-015-0362-4)
Supplement: Supplementary file 2 — 10.1186/s13068-015-0362-4 In the Supplemental Material Section results of regression analysis of quadratic response surface model fitting in the optimization of components for LiP synthesis are presented. [file 13068_2015_362_MOESM2_ESM.docx]

**Zhang et al. Additional file Table 2: In the Supplementa Material Section results of regression analysis of quadratic response surface model fitting in the optimization of components for LiP synthesis are presented.**

| Source | Sum of Squares | df | Mean Square | F-Value | p-value  Prob > F |
| --- | --- | --- | --- | --- | --- |
| Model | 1097281 | 20 | 54864 | 12.14 | < 0.0001^a^ |
| X_1_-glucose | 12656 | 1 | 12656 | 2.799 | 0.1068 |
| X_2_-sodium nitrate | 26001 | 1 | 26001 | 5.752 | 0.0243 |
| X_3_-corn steep liquor | 240.3 | 1 | 240.3 | 0.053 | 0.8196 |
| X_4_-yeast extract | 2756 | 1 | 2756 | 0.610 | 0.4422 |
| X_5_-1 vitamin | 20093 | 1 | 20093 | 4.445 | 0.0452 |
| X_1_X_2_ | 9409 | 1 | 9409 | 2.081 | 0.1615 |
| X_1_X_3_ | 19182 | 1 | 19182 | 4.243 | 0.0500 |
| X_1_X_4_ | 96100 | 1 | 96100 | 21.26 | 0.0001 |
| X_1_X_5_ | 8372 | 1 | 8372 | 1.852 | 0.1857 |
| X_2_X_3_ | 25 | 1 | 25 | 0.006 | 0.9413 |
| X_2_X_4_ | 40602 | 1 | 40602 | 8.982 | 0.0061 |
| X_2_X_5_ | 50176 | 1 | 50176 | 11.10 | 0.0027 |
| X_3_X_4_ | 11990 | 1 | 11990 | 2.652 | 0.1159 |
| X_3_X_5_ | 43681 | 1 | 43681 | 9.663 | 0.0046 |
| X_4_X_5_ | 8281 | 1 | 8281 | 1.832 | 0.1880 |
| X_1_^2^ | 673266 | 1 | 673266 | 148.9 | < 0.0001 |
| X_2_^2^ | 233299 | 1 | 233299 | 51.61 | < 0.0001 |
| X_3_^2^ | 79178 | 1 | 79178 | 17.52 | 0.0003 |
| X_4_^2^ | 60970 | 1 | 60970 | 13.49 | 0.0011 |
| X_5_^2^ | 63798 | 1 | 63798 | 14.11 | 0.0009 |
| Residual | 113013 | 25 | 4520 |  |  |
| Lack of Fit | 103570 | 20 | 5178 | 2.742 | 0.1334 |
| Pure Error | 9443 | 5 | 1888 |  |  |
| Cor Total | 1210295 | 45 |  |  |  |

Cor Total 45, R^2^ =0.9066.

^a^Values of ‘probability> F’ less than 0.05 indicate significant model terms.
