# Supplementary material for: Lignin degradation in corn stalk by combined method of H2O2 hydrolysis and Aspergillus oryzae CGMCC5992 liquid-state fermentation
Source: Biotechnol Biofuels. 2015 Nov 19;8:183. doi: 10.1186/s13068-015-0362-4 (PMC4653895; doi:10.1186/s13068-015-0362-4)
Supplement: Supplementary file 3 — 10.1186/s13068-015-0362-4 In the Supplemental Material Section Box–Behnken design and the result in the optimization of fermentation broth amount H2O2 concentration, H2O2 amount and H2O2 flow rate are presented. [file 13068_2015_362_MOESM3_ESM.docx]

**Zhang et al. Additional file Table 3: In the Supplementa Material Section Box-Behnken design and the result in the optimization of fermentation broth amount H2O2 concentration, H2O2 amount and H2O2 flow rate are presented.**

| Run | Fermentation broth amount (mL) | H_2_O_2_ concentration (%) | H_2_O_2_ flowing rate (mL/min) | H_2_O_2_ volume (mL) | Sugar yield (%) |
| --- | --- | --- | --- | --- | --- |
| 1 | 0 | 0 | -1 | 1 | 22.06 |
| 2 | -1 | 0 | 1 | 0 | 11.21 |
| 3 | 0 | 0 | 1 | 1 | 20.63 |
| 4 | 1 | 0 | -1 | 0 | 16.83 |
| 5 | 0 | 0 | 1 | -1 | 15.73 |
| 6 | 0 | -1 | 1 | 0 | 13.16 |
| 7 | -1 | -1 | 0 | 0 | 11.19 |
| 8 | -1 | 1 | 0 | 0 | 15.04 |
| 9 | 0 | 1 | 1 | 0 | 19.58 |
| 10 | 0 | 0 | 0 | 0 | 16.56 |
| 11 | 0 | 0 | 0 | 0 | 16.47 |
| 12 | 1 | 0 | 0 | -1 | 20.98 |
| 13 | 1 | -1 | 0 | 0 | 21.28 |
| 14 | 0 | 1 | -1 | 0 | 12.06 |
| 15 | 0 | -1 | 0 | -1 | 14.01 |
| 16 | 0 | -1 | 0 | 1 | 19.92 |
| 17 | 0 | 0 | -1 | -1 | 14.93 |
| 18 | -1 | 0 | -1 | 0 | 11.65 |
| 19 | -1 | 0 | 0 | -1 | 13.24 |
| 20 | 0 | 0 | 0 | 0 | 18.64 |
| 21 | 1 | 0 | 1 | 0 | 22.37 |
| 22 | 0 | 0 | 0 | 0 | 16.38 |
| 23 | 1 | 0 | 0 | 1 | 24.71 |
| 24 | 1 | 1 | 0 | 0 | 19.14 |
| 25 | 0 | 1 | 0 | -1 | 15.14 |
| 26 | 0 | 1 | 0 | 1 | 18.47 |
| 27 | -1 | 0 | 0 | 1 | 16.35 |
| 28 | 0 | 0 | 0 | 0 | 16.53 |
| 29 | 0 | -1 | -1 | 0 | 15.87 |
