# Supplementary material for: Lignin degradation in corn stalk by combined method of H2O2 hydrolysis and Aspergillus oryzae CGMCC5992 liquid-state fermentation
Source: Biotechnol Biofuels. 2015 Nov 19;8:183. doi: 10.1186/s13068-015-0362-4 (PMC4653895; doi:10.1186/s13068-015-0362-4)
Supplement: Supplementary file 4 — 10.1186/s13068-015-0362-4 In the Supplemental Material Section Box–Behnken design and the result in the optimization of the conditions of hydrolysis reaction are presented. [file 13068_2015_362_MOESM4_ESM.docx]

**Zhang et al. Additional file Table 4: In the Supplementa Material Section Box-Behnken design and the result in the optimization of the conditions of hydrolysis reaction are presented.**

| Run | water material ratio | hydrolysis temperature (^o^C) | pretreatment hydrolysis temperature (^o^C) | pretreatment time (min) | Sugar yield （%） |
| --- | --- | --- | --- | --- | --- |
| 1 | -1 | 0 | 0 | 1 | 42.39 |
| 2 | 1 | 0 | 0 | 1 | 37.59 |
| 3 | 0 | 0 | -1 | 1 | 40.68 |
| 4 | 1 | 0 | -1 | 0 | 31.98 |
| 5 | -1 | 0 | 0 | -1 | 28.72 |
| 6 | 0 | 1 | 0 | 1 | 36.18 |
| 7 | 0 | -1 | 0 | 1 | 32.28 |
| 8 | 0 | 0 | 0 | 0 | 42.53 |
| 9 | 0 | 0 | 1 | -1 | 36.18 |
| 10 | 0 | -1 | -1 | 0 | 32.15 |
| 11 | -1 | 0 | 1 | 0 | 39.98 |
| 12 | 0 | 1 | 0 | -1 | 27.72 |
| 13 | 0 | 1 | 1 | 0 | 36.18 |
| 14 | 0 | 0 | 0 | 0 | 41.57 |
| 15 | 0 | 0 | 0 | 0 | 43.32 |
| 16 | 0 | -1 | 1 | 0 | 39.81 |
| 17 | 0 | 0 | 1 | 1 | 37.98 |
| 18 | 0 | 0 | 0 | 0 | 42.51 |
| 19 | 0 | 0 | 0 | 0 | 45.21 |
| 20 | -1 | 0 | -1 | 0 | 31.92 |
| 21 | 1 | 1 | 0 | 0 | 34.73 |
| 22 | -1 | 1 | 0 | 0 | 33.48 |
| 23 | 1 | 0 | 1 | 0 | 39.33 |
| 24 | 1 | 0 | 0 | -1 | 30.05 |
| 25 | 1 | -1 | 0 | 0 | 37.79 |
| 26 | 0 | 1 | -1 | 0 | 35.19 |
| 27 | 0 | -1 | 0 | -1 | 28.59 |
| 28 | -1 | -1 | 0 | 0 | 40.11 |
| 29 | 0 | 0 | -1 | -1 | 25.28 |
