# Supplementary material for: Lignin degradation in corn stalk by combined method of H2O2 hydrolysis and Aspergillus oryzae CGMCC5992 liquid-state fermentation
Source: Biotechnol Biofuels. 2015 Nov 19;8:183. doi: 10.1186/s13068-015-0362-4 (PMC4653895; doi:10.1186/s13068-015-0362-4)
Supplement: Supplementary file 5 — 10.1186/s13068-015-0362-4 In the Supplemental Material Section results of regression analysis for quadratic response surface model fitting (ANOVA) in the optimization of enzyme amount and H2O2 flow rate are presented. [file 13068_2015_362_MOESM5_ESM.docx]

**Zhang et al. Additioal file Table 5: In the Supplementa Material Section results of regression analysis for quadratic response surface model fitting (ANOVA) in the optimization of enzyme amount and H2O2 flow rate are presented.**

| Source | Sum of  Squares | df | Mean Square | F-Value | p-value of  Prob > F |
| --- | --- | --- | --- | --- | --- |
| Model | 338.7 | 14 | 24.19 | 16.12 | < 0.0001 |
| X_1_-enzyme volume | 181.2 | 1 | 181.2 | 120.8 | < 0.0001 |
| X_2_-H_2_O_2_ concentration | 1.333 | 1 | 1.333 | 0.889 | 0.3618 |
| X_3_-H_2_O_2_ flow rate | 7.177 | 1 | 7.177 | 4.783 | 0.0462 |
| X_4_- H_2_O_2_ volume | 65.85 | 1 | 65.85 | 43.88 | < 0.0001 |
| X_1_X_2_ | 8.970 | 1 | 8.970 | 5.978 | 0.0283 |
| X_1_X_3_ | 8.940 | 1 | 8.940 | 5.958 | 0.0285 |
| X_1_X_4_ | 0.096 | 1 | 0.096 | 0.064 | 0.8039 |
| X_2_X_3_ | 26.16 | 1 | 26.16 | 17.44 | 0.0009 |
| X_2_X_4_ | 1.664 | 1 | 1.664 | 1.109 | 0.3101 |
| X_3_X_4_ | 1.243 | 1 | 1.2435 | 0.829 | 0.3781 |
| X_1_^2^ | 0.132 | 1 | 0.132 | 0.088 | 0.7708 |
| X_2_^2^ | 6.466 | 1 | 6.466 | 4.309 | 0.0568 |
| X_3_^2^ | 4.642 | 1 | 4.642 | 3.093 | 0.1004 |
| X_4_^2^ | 17.99 | 1 | 17.99 | 11.99 | 0.0038 |
| Residual | 21.01 | 14 | 1.501 |  |  |
| Lack of Fit | 17.27 | 10 | 1.727 | 1.850 | 0.2901 |
| Pure Error | 3.734 | 4 | 0.934 |  |  |
| Cor Total | 359.7 | 28 |  |  |  |

R^2^ = 0.9416
