# Supplementary material for: Lignin degradation in corn stalk by combined method of H2O2 hydrolysis and Aspergillus oryzae CGMCC5992 liquid-state fermentation
Source: Biotechnol Biofuels. 2015 Nov 19;8:183. doi: 10.1186/s13068-015-0362-4 (PMC4653895; doi:10.1186/s13068-015-0362-4)
Supplement: Supplementary file 6 — 10.1186/s13068-015-0362-4 In the Supplemental Material Section the results of regression analysis for quadratic response surface model fitting (ANOVA) in the optimization of the conditions of hydrolysis reaction are present. [file 13068_2015_362_MOESM6_ESM.docx]

**Zhang et al. Additional file Table 6: In the Supplementa Material Section the results of regression analysis for quadratic response surface model fitting (ANOVA) in the optimization of the conditions of hydrolysis reaction are present.**

| Source | Sum of squares | df | Mean square | F-value | p-value of  prob > F |
| --- | --- | --- | --- | --- | --- |
| Model | 708.8 | 14 | 50.62 | 9.913 | < 0.0001 |
| X_1_-water/material ratio | 2.193 | 1 | 2.193 | 0.429 | 0.5229 |
| X_2_-hydrolysis temperature | 4.380 | 1 | 4.380 | 0.858 | 0.3701 |
| X_3_-pretreatment temperature | 86.73 | 1 | 86.73 | 16.98 | 0.0010 |
| X_4_- pretreatment time | 213.0 | 1 | 213.0 | 41.71 | < 0.0001 |
| X_1_X_2_ | 3.186 | 1 | 3.186 | 0.624 | 0.4428 |
| X_1_X_3_ | 0.1260 | 1 | 0.126 | 0.025 | 0.8774 |
| X_1_X_4_ | 9.394 | 1 | 9.394 | 1.840 | 0.1965 |
| X_2_X_3_ | 11.12 | 1 | 11.12 | 2.178 | 0.1621 |
| X_2_X_4_ | 5.688 | 1 | 5.688 | 1.114 | 0.3091 |
| X_3_X_4_ | 46.24 | 1 | 46.24 | 9.054 | 0.0094 |
| X_1_^2^ | 52.71 | 1 | 52.71 | 10.32 | 0.0063 |
| X_2_^2^ | 136.3 | 1 | 136.3 | 26.68 | 0.0001 |
| X_3_^2^ | 59.43 | 1 | 59.43 | 11.64 | 0.0042 |
| X_4_^2^ | 226.1 | 1 | 226.1 | 44.28 | < 0.0001 |
| Residual | 71.50 | 14 | 5.107 |  |  |
| Lack of Fit | 64.01 | 10 | 6.401 | 3.419 | 0.1237 |
| Pure Error | 7.488 | 4 | 1.872 |  |  |
| Cor Total | 780.2 | 28 |  |  |  |

R^2^ = 0.9084
